# Imaging of EGFR and EGFR Tyrosine Kinase Overexpression in Tumors by Nuclear Medicine Modalities

**DOI:** 10.2174/138161208786404326

**Published:** 2008-10

**Authors:** Eyal Mishani, Galith Abourbeh, Martin Eiblmaier, Carolyn J Anderson

**Affiliations:** 1 Department of Nuclear Medicine, Cyclotron Unit, Hadassah Hebrew University Hospital, Jerusalem, Israel; 2 Mallinckrodt Institute of Radiology, Washington University School of Medicine, St. Louis, MO; 3 Department of Chemistry, Washington University, St. Louis, MO, USA

**Keywords:** EGFR, PET, cancer, imaging, tyrosine kinase, cetuximab, gefitinib.

## Abstract

Protein tyrosine kinases (PTKs) play a pivotal role in signal transduction pathways and in the development and maintenance of various cancers. They are involved in multiple processes such as transcription, cell cycle progression, proliferation, angiogenesis and inhibition of apoptosis. Among the PTKs, the EGFR is one of the most widely studied and has emerged as a promising key target for the treatment of cancer. Indeed, several drugs directed at this receptor are FDA-approved and many others are at various stages of development. However, thus far, the therapeutic outcome of EGFR-targeted therapy is suboptimal and needs to be refined. Quantitative PET molecular imaging coupled with selective labelled biomarkers may facilitate *in vivo *EGFR-targeted drug efficacy by noninvasively assessing the expression of EGFR in tumor, guiding dose and regime by measuring target drug binding and receptor occupancy as well as potentially detecting the existence of a primary or secondary mutation leading to either drug interaction or failure of EGFR recognition by the drug. This review describes the attempts to develop labelled EGFR molecular imaging agents that are based either on low molecular weight tyrosine kinase inhibitors or monoclonal antibodies directed to the extracellular binding domain of the receptor to be used in nuclear medicine modalities.

## INTRODUCTION

In 2005, deaths attributed to cancer reached 7.5 millions worldwide, and the number of diagnosed cases is postulated to gradually increase and reach about 20 million in 2020 [1]. The battle to combat this deadly disease was initiated in the late 1940's with the introduction of the first chemotherapeutic agents. These non-specific agents predominantly affect hyper-proliferating cells, characteristic of many cancers, and exert their cytotoxic effects through different pharmacological modes of action, including induction of DNA damage, inhibition of RNA and DNA synthesis, and/or disruption of the cytoskeleton. The significant contribution to cancer management due to the institution of such chemotherapeutic agents cannot be overestimated. Nonetheless, the overall toxicities and side effects of these drugs limit the dose regime resulting in a narrow therapeutic index and, in most cases the produced responses are insufficient and unpredictable. Furthermore, during the course of therapy, cross-resistance to different chemotherapeutic drugs can be associated with recurrence of the disease [2-4]. 

In the late 1980's, improved and more specific approaches for treating cancer emerged by virtue of a plethora of chemical and biochemical technologies. One such valid approach involves various strategies that specifically interfere with crucial signaling pathways which are dysregulated in malignant cells and play a pivotal role in the development and maintenance of various cancers. Much effort within this approach has focused on the inhibition of protein tyrosine kinases (PTKs). There are approximately 20 classes of PTKs, including the epidermal growth factor (EGF), insulin, PDGF, FGF, VEGF, and HGF receptor families [5]. The EGF family (receptor tyrosine kinase class I) of membrane receptors, also called human epidermal receptor (HER) family, is one of the most relevant targets in this class. There are four closely related receptors in this family [6]: EGFR (HER1, *erb*B1), HER2 (*neu*, *erb*B2), HER3 (*erb*B3), and HER4 (*erb*B4). Ligands include EGF, amphiregulin, and TGF-α for EGFR, and a group of differentiation factors called neuregulins for HER3 and HER4. Heparin-binding EGF, betacellulin, and epiregulin can stimulate EGFR and HER4. In all cases, ligand binding is followed by formation of receptor homo- or heterodimers. HER2 is unique in that it has no specific ligand and is locked constitutively in a conformation resembling the ligand-bond states. As such, it is subject to heterodimerization with other members of the activated HER family.

## EPIDERMAL GROWTH FACTOR RECEPTOR (EGFR)

EGFR (170 kDa) is a membrane-spanning glycoprotein and consists of an extracellular ligand-binding domain, a transmembrane domain, and an intracellular domain with tyrosine kinase activity. Binding of ligand induces the dimerization of receptors, followed by activation of the cytoplasmic tyrosine kinase domain and the subsequent signal transduction cascade. Signal termination occurs mainly by endocytosis of the activated receptor [7, 8]. EGFR is associated with oncogenic transformation, and dysregulation of EGFR is associated with all of the key features of cancer [8]. An increased expression of EGFR is the hallmark of many human tumors such as breast cancer, squamous cell carcinoma of the head and neck, and prostate cancer (Table **1**).

## PTKs AS TARGETS FOR CANCER THERAPY

PTKs catalyze the transfer of phosphate in ATP to specific tyrosine residues within proteins, thereby altering their structure and function. They play a crucial role in signal transduction pathways that control both intracellular signaling and intercellular communication and are involved in multiple processes such as metabolism, transcription, cell cycle progression, cytoskeletal rearrangement and cell movement, proliferation, angiogenesis and inhibition of apoptosis. Of the 91 PTKs identified thus far, 59 are receptor TKs, and 32 are cellular TKs, most of them regulating signaling which is crucial for normal cell development and survival. Since PTKs hold a key position in the function of multicellular organisms, it is not surprising that PTK malfunction can lead to conditions such as psoriasis, cardiovascular disease, atherosclerosis and cancer. Most diseases involve PTK dysregulation owing to: a) overexpression of TK receptor and its ligand or both resulting in autocrine-paracrine stimulation; b) dimerization of a PTK receptor or a cellular PTK with a related protein; c) various mutations in the PTK itself, leading to enhanced and sometimes persistent stimulation [9-12]. Approximately 50% of the known oncogenes encode PTKs [13, 14]. As a result, PTKs, whether receptors or cellular proteins have become valid targets to combat cancer through specific biochemical mechanisms meant to decrease side effects [15-25]. 

**Fig. (1):**
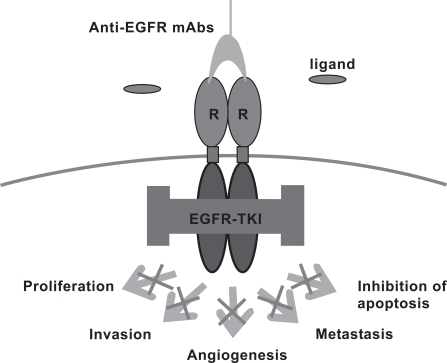
Mechanism of action of anti-EGFR mAb-based drugs.

Receptor PTKs can be targeted by two classes of drugs [26]: in the first class, monoclonal antibodies (mAbs) target the extracellular binding domain (Fig. **1**) [27], and inhibit cancer cell growth *via* several direct processes such as interrupting PTK signaling in most cases by blockage of their ligand binding, inhibiting cell cycle progression or DNA repair, reducing angiogenesis, and inducing apoptosis, and *via* indirect processes mediated by the immune system such as complement-dependent cytotoxicity (CDC) and antibody-dependent cellular cytotoxicity (ADCC) [26] . In the second class, low molecular weight TK inhibitors target the intracellular ATP binding domain of the receptor [15]. Cellular PTKs are exclusively targeted by the latter class of drugs. One of the first PTK mAb inhibitors is the IgG_1_-class monoclonal antibody trastuzumab (Herceptin^®^) (Table **2**). Trastuzumab is an anti-erbB2 mAb which increases response rates and improves survival in patients with erbB2-overexpressing breast cancer when combined with conventional chemotherapy [28, 29]. It is unique in that, unlike mAbs which target the EGFR, it is only active against cancers that overexpress its target, therefore erbB2 testing prior to treatment is mandatory. Cetuximab is another IgG_1_ class mAb targeting the EGFR (Table **2**). Surprisingly, although this mAb displays insufficient effect against EGFR-overexpressing breast cancer, it has yielded positive clinical results against head and neck cancers overexpressing the EGFR and has demonstrated activity in colon cancer regardless of tumor EGFR expression [30-32]. The mechanisms of action of Cetuximab remain unknown, yet more disturbingly, is the inability to predict patient response. Panitumumab and Matuzumab are other anti-EGFR mAbs (Table **2**). Panitumumab has a moderate activity in primary and metastatic colorectal carcinoma with no evident correlation with tumor EGFR expression [33-35]. The clinical results of Matuzumab in colorectal cancer are suboptimal and the drug is currently undergoing phase II clinical trials for the treatment of lung and stomach cancer. Bevacizumab is an anti VEGF mAb which is used to suppress tumor growth through inhibition of angiogenesis. This drug was approved by the FDA for use in combination with standard chemotherapy in the treatment of metastatic colon cancer and all forms of metastatic non small cell lung cancer. Lately, it was approved by the FDA for the treatment of breast cancer. Although some of the above mentioned mAbs have become accepted drugs in clinical practice, currently there is no reliable clinical modality that would properly select responders and predict therapeutic outcome. However, a correlation between tumor response and skin toxicity has been found which may indicate that some patients not responding to treatment were "underdosed" [36, 37].

The second class of compounds is low molecular weight TK inhibitors which target the ATP binding domain of PTKs and disrupt the hyperactive signal transduction pathways in cancer cells. The first step in the development of low molecular weight PTK inhibitors began shortly following the recognition in the early 1980's that natural compounds such as quercetin, genistein, lavendustin, erbstatin and herbimycin A [38-43] are potent inhibitors of PTKs. Although these natural compounds have no selectivity and therefore were found to be very toxic, they served as starting templates for the design and development of synthetic, more potent and selective PTK inhibitors. The benzylidine moiety of erbstatin and other arylidene derivatives were developed into a class of PTK blockers defined as tyrphostins (tyrosine phosphorilation). The first group of PTK inhibitors synthesized was the benzene malononitrile tyrphostins [44-47]. These compounds were competitive with the substrate and non competitive with ATP. In the mid-1990's, when structure activity led to bicyclic tyrphostins, the main thrust in the development of PTK inhibitors was to endorse a generation of ATP competitive kinase inhibitors. The most common chemical structures of these tyrphostins are anilinoquinazolines, anilinoquinolines and anilinopyridopirimidines. 

**Fig. (2):**
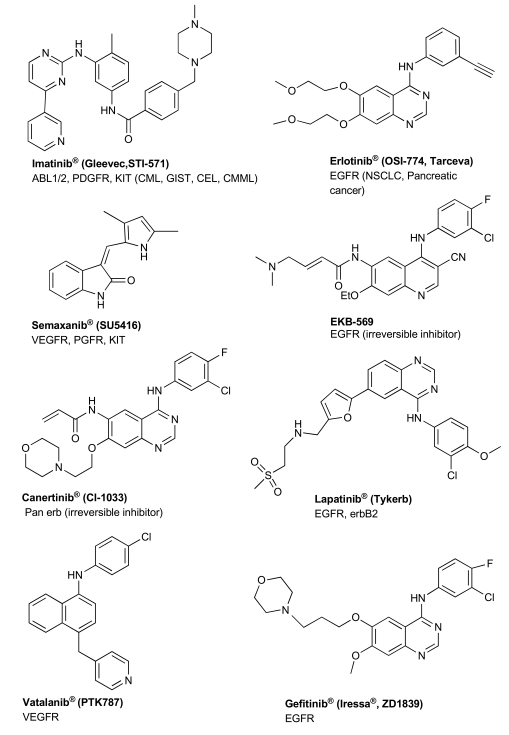
Chemical structure of PTKS drugs

Although ATP binding sites are highly conserved among tyrosine kinases, minor differences in kinase domain structures in combination with combinatorial chemistry, structured-based drug design and computational chemistry have led to the development of highly selective PTK inhibitors. In the past few years, over 30 inhibitors were at various stages of development. Clinical studies conducted recently have recognized some of these inhibitors as therapeutic drugs for specific cancers. Imatinib (STI-571;Gleevec) (Table **2**, Fig. **2**) has been identified as an effective Bcr-Abl inhibitor with durable response in patients in the early phase of chronic myelogenous leukemia (CML) with minimal side effects [48, 49]. Imatinib is also the standard of care, first-line treatment for unresectable or metastatic gastrointestinal stromal tumors [GIST] [50], especially in patients who harbor activating mutations in c-Kit. 

In the field of cancer angiogenesis, various VEGFR inhibitors are currently being evaluated in clinical trials, including semaxanib and vatalanib (Table **2**, Fig. **2**). In clinical studies, vatalanib, which inhibits VEGFR-1 and -2, PDGFR and c-Kit, has been shown to suppress hepatocellular carcinoma (HCC) growth and to have anti-neoplastic effects in other solid tumors [51, 52]. Sunitinib (Sutent, Table **2**), another promising agent against angiogenesis, inhibits the VEGFR as well as the PDGFR, c-KIT and Flt3 tyrosine kinases. Sunitinib has been approved for the treatment of renal cell carcinoma and for the treatment of GIST, and is currently tested in phase II trials for HCC [53, 54]. Most of these drugs produce limited response as monotherapeutic agents against solid tumors and are considered to be more effective in combination with other PTK drugs [55-59]. 

Three EGFR/erbB2 low molecular weight reversible inhibitors have thus far been approved for clinical use: lapatinib, gefitinib and erlotinib (Table **2**, Fig. **2**), and numerous other drug candidates are in various stages of development. Gefitinib and erlotinib are more selective for the EGFR, while lapatinib targets both EGFR and erbB2. Promising results have been obtained using gefitinib and erlotinib in pre-clinical models of EGFR overexpressing cell lines and xenografts [60, 61]. Nonetheless, these results failed to reproduce in the clinical setting since both agents appear to be effective only in the subset of NSCLC patients [62]. Higher response rates to gefitinib and erlotinib therapies have also been reported in patients with EGFR expressing tumors containing well-defined activating mutations [63-67]. This higher sensitivity of the mutant form could be due to structural changes at the kinase domain mutation rendering the receptor more receptive to inhibitor binding. However, similarly to imatinib, patients who initially responded to these treatments have occasionally developed therapy resistance due to acquired secondary mutation in the EGFR [68], thus, necessitating the acquisition of further clinical and experimental data so as to better predict response to such therapies. More recent pre-clinical and clinical publications indicate that irreversible inhibitors of the EGFR such as Canertinib and EKB-569 (Fig. **2**, Table **2**) appear to circumvent this resistance suggesting that this category of inhibitors may benefit from a larger clinical application [68-72]. 

The concept of targeted therapies by specifically inhibiting PTKs in cancer is very promising, albeit several drugs that have entered clinical trials have not yielded the predicted positive outcome. This inconsistency can be attributed to several factors working in unison or independently of each other including: inappropriate selection of potential responders in terms of the expression of the targeted protein in a particular tumor; insufficient inhibition of the targeted PTKs due to inappropriate dosage schedule; resistance due to a secondary mutation in the PTKs developed during therapy; loss of the survival factor role of the targeted PTKs in cancer cells during therapy, and the development of other pathways as a salvage and escape mechanism for cancer cell survival. One possible approach that may yield better results would be either to use multi-drug therapies working in synergy and meant to target several PTKs [15, 73], or drugs targeting multiple PTKs which play a major role in cancer cell proliferation. Better yet, a reliable and accurate quantitative assay to determine PTK levels and their role in tumors is required to establish a customized targeted treatment [74]. 

## NUCLEAR MEDICINE AND PET MOLECULAR IMAGING OF EGFR

As tumor cells are characterized by uncontrolled proliferation and enhanced cell growth, it has been observed that most tumor cells over-express one or several hormone receptors [75]. Radiopharmaceuticals which selectively target those receptors can be used to diagnose and/or treat cancer. Molecular imaging of tumors *via* nuclear medicine modalities such as single photon computed tomography (SPECT) or positron emission tomography (PET) using targeted radiopharmaceuticals, could visualize the underlying mechanism of cellular processes *in vivo* and complements and enhances anatomical information acquired by computed tomography (CT) and magnetic resonance imaging (MRI). PET is based on the administration of radioactively-labeled probes (radiopharmaceutical) with characteristic physiological or biological properties. Following the administration of the probe, spatial and temporal monitoring of its biodistribution within the body is conducted using a PET scanner. To this end, the desired molecule (either natural or synthetic) is labeled with a positron-emitting isotope. Positrons (*β^+^) *travel only a few millimeters before they encounter an electron, which leads to the formation of two annihilation photons at a 180° angle. Simultaneous detection of these two 511 keV photons by the scanner forms the basis of PET imaging. The resolution of clinical PET scanners is in the low millimeter range, which is not as high as MRI (100–500 μm), but higher than that of the alternative method SPECT (3–6 mm). However modern PET/ CT scanners offer a more accurate anatomic localization of radioactivity, thereby enhancing the interpretation of PET images. The superiority of nuclear medicine compared to other imaging modalities stems from its high sensitivity, in a nanomolar (nM) concentration-range, as opposed to millimolar (mM) concentrations for other related imaging techniques, thus enabling *in vivo* quantitative visualization of "low capacity systems" such as receptors and enzymes. After a tumor is diagnosed, a targeted radiopharmaceutical can be used to determine the optimal therapy by identifying key molecular markers on the tumor cells. During the course of therapy, the radiopharmaceutical can be used to monitor early response to the chosen targeted treatment.

Immunohistochemistry (IHC) is the most frequently applied method for evaluating PTK expression in tumor tissues, however, it requires tissue biopsies, which are not always available and furthermore, do not always represent the pathology of the whole tumor nor of distant, unexamined metastases. Additionally, IHC provides only semi-quantitative data, and can be inconsistent due to variations in methodology [76]. Thus, many hurdles remain to be overcome in order to effectively treat various types of cancers by targeted PTK therapy. Molecular imaging such as PET coupled with suitable selective labeled bioprobes that target specific PTKs has the potential to resolve some of the above mentioned obstacles by: 1) noninvasively determining whether the target protein is expressed in a specific tumor and its metastasis; 2) monitoring target-drug binding and receptor occupancy *in vivo*; 3) determining duration of PTK inhibition *in vivo*; and 4) potentially identifying the existence of a primary or secondary mutation leading to either drug interaction or loss of PTK recognition by the drug.

As demonstrated above, EGFR expression in tumors is not sufficient to predict EGFR targeted therapeutic response. However, in many cases, its overexpression in tumors is a prerequisite for initiating such treatment and can be measured non-invasively by molecular imaging modalities such as PET. Indeed, within the PTKs, most research has primarily focused on EGFR as a target for PET imaging either by the development and acquisition of labeled monoclonal antibodies directed to the extracellular binding domain or by the development of low molecular weight PET agents derived from the anilinoquinazoline skeleton of existing or potential drugs targeting the intracellular ATP binding domain of the receptor [77].

## RADIOPHARMACEUTICALS FOR IMAGING EGFR EXPRESSION IN TUMORS

### Radiolabeled Intact mAbs for Imaging EGFR Expression in Tumors

The use of radiolabeled anti-EGFR antibodies for EGFR-expressing cancer diagnosis has become the subject of intense investigation as more mAbs with relevant and well-characterized specificities become available. Anti-EGFR mAbs have been used and evaluated for imaging with a variety of radionuclides, including radiometals and radiohalogens (Table **3**). Cetuximab attached to the radiometal chelator diethylenetriaminepentaacetic acid (DTPA) and labeled with ^111^In was shown to localize specifically in tumors that over-express EGFR [78, 79]. However, a considerable amount of radioactivity in the liver was observed limiting the clinical usefulness of this agent for cancer imaging. An improved cetuximab conjugate (DTPA-PEG-cetuximab) was reported to overcome this problem, as tumor imaging of ^111^In-DTPA-PEG-cetuximab in nude mice showed significant reduction of radioactivity in the liver using a gamma camera [79]. Although cetuximab Fab' and F(ab')_2_ fragments have been investigated, they have reduced binding affinity (5-fold weaker) and showed less inhibition of tumor growth than the intact antibody [80]. 

Perk *et al.*, reported the biodistribution of positron-emitting ^89^Zr-deferrioxamine-cetuximab as a surrogate imaging agent for therapy with ^90^Y and/or ^177^Lu-DOTA-cetuximab [81]. Cetuximab-*N*-sucDf-^89^Zr showed very comparable tumor uptake and clearance in non-target tissues to cetuximab-*p*-SCN-Bz-DTPA-^88^Y (where gamma-emitting ^88^Y was used for biodistribution studies) and cetuximab-*p*-SCN-Bz-DTPA-^177^Lu. The only difference in biodistribution was a higher uptake of cetuximab-*N*-sucDf-^89^Zr in the bone, compared to the ^88^Y and ^177^Lu-labeled agents.

**Fig. (3):**
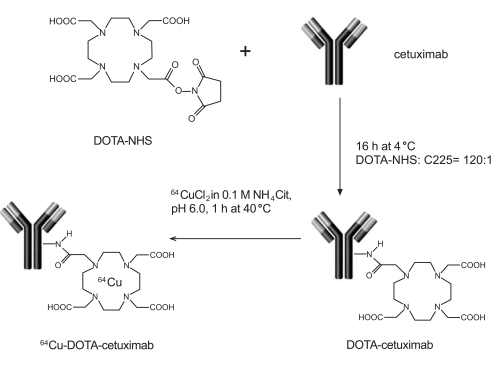
Preparation of ^64^Cu-DOTA-cetuximab for PET imaging of EGFR-positive tumors.

Cai *et al.*, reported the biodistribution of ^64^Cu-DOTA-cetuximab (Fig. **3**) in seven different EGFR-expressing tumor-bearing mouse models [82]. A correlation between EGFR expression by Western blot analysis and %ID/g in the various tumor types was observed, suggesting that ^64^Cu-DOTA-cetuximab is a potentially accurate biomarker for EGFR expression. In another study, Li* et al.* showed high uptake of ^64^Cu-DOTA-cetuximab in A431 tumors, although there was significant uptake in the liver, in part due to ^64^Cu dissociation from the DOTA chelator [83]. Improved chelation systems for labeling ^64^Cu to cetuximab will greatly improve this agent for future human PET imaging studies. 

### Radiolabeled Affibodies for Imaging EGFR Expression

Smaller molecular weight “affibodies” that bind to EGFR have been developed as alternatives to radiolabeled intact mAbs as imaging agents. Affibody molecules are three-helix bundle molecules based on 58 amino acids and are derived from the IgG-binding domains of staphylococcal protein A [84]. (Z_EGFR: 955_)_2_, a 14.6 kDa molecule, was selected from a phage display library consisting of 13 randomized residues, and binds to EGFR on cultured cells with low nanomolar affinity [85]. The smaller size compared to intact antibodies (~150 kDa) will allow greater tumor penetration and more rapid blood clearance.

The Affibody (Z_EGFR: 955_)_2_ was labeled with ^125^I and compared to ^125^I-labeled EGF and cetuximab [84]. In A431 cells grown in culture, the uptake of ^125^I-labeled (Z_EGFR: 955_)_2_ was more rapid than that of the other agents, and was found to be cell-associated for a longer time period, suggesting more rapid internalization and greater retention in EGFR-positive tumor cells. Affibody (Z_EGFR: 955_)_2_ was also labeled with ^111^In, and in A431 tumor-bearing mice showed high tumor uptake, with tumor: blood ratios of 9.1 at 4 h post-injection [86].

Additional Affibody molecules were developed through the affinity maturation process and radiolabeled with ^111^In [87] for evaluation in A431 tumor-bearing mice. All agents evaluated in tumor-bearing mice showed tumor uptake between 5-7% ID/g at 4 h post-injection with tumor: blood ratios of ~3. The kidney uptake was extremely high, however, with uptake ranging between 100-200 %ID/g. 

### Low Molecular Weight Imaging Agents

**Fig. (4):**
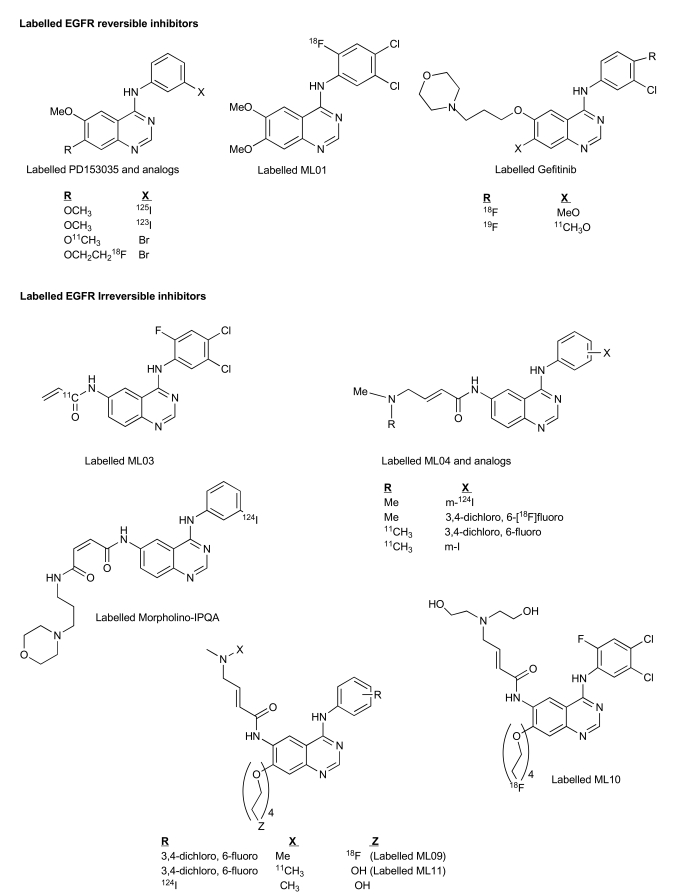
Labelled EGFR bioprobes.

Attempts to develop low-molecular weight imaging agents that target the TK domain of the EGFR have been mainly focused on the 4-anilinoquinazoline class of compounds that has been originally developed for therapy. The reversible inhibitor PD 153035 was one of the first templates used for developing such molecular imaging agents. It was labeled with carbon-11 at the 7-methoxy position of the quinazoline ring (Fig. **4**) and its biodistribution properties and specific uptake in the examined tissues have been investigated in non-tumor bearing mice [88]. Specific binding in these blocking studies by pre-administration of an excess of unlabeled PD153035 could not be demonstrated and an increase (rather than a reduction) in the levels of activity uptake in tissues was obtained [88]. In another study, PD153035 was non-specifically labeled either at the 6- or 7-methoxy positions and its biodistribution using PET in rats bearing EGFR-rich SH-SY5Y human neuroblastoma xenografts, revealed a low maximum tumor activity-uptake value of ~0.3% injected tracer dose per mL of tumor tissue (%ID/mL) at less than 10 min post injection [89]. At this time period, radioactivity uptakes in the gastrointestinal (GI) tract and in the liver were about 2 to 2.5-fold higher, resulting in high activity background levels. Moreover, specific tumor uptake was not confirmed in a blocking study. Derivatives of PD 153035 were labeled with ^123^I (t_1/2_ = 13.3 h), ^125^I (t_1/2_ = 59.4 d) and ^18^F (t_1/2_ = 110 min.) (Fig. **4**); however, specific binding of the labeled compounds to the EGFR was demonstrated only *in vitro* using EGFR positive MDA-MB 468 human breast cancer cells [90]. In a later report, the biodistribution of ^123^I-PD153035 analog was evaluated by a dynamic gamma camera scan in rats bearing subcutaneous xenografts of 13762 MAT rat mammary adenocarcinoma cells [91]. Although it remains unclear whether this cell line expresses the EGFR, maximal tumor activity uptake was obtained between 30 and 60 min post injection of the tracer, followed by a decrease to background tissue levels after 5 h. Quantification of the data was neither performed, nor was there any specific binding demonstrated in this study.

A radiosynthetic strategy has been developed for fluorine-18 labelling of 6,7-disubstituted 4-anilinoquinazolines at the anilino moiety [92-94]. In general, it was based on the nucleophilic substitution of the nitro group of dinitrobenzene derivatives with fluorine-18, followed by reduction of the second nitro group to yield labeled aniline. Subsequently, the [^18^F]-labeled aniline was coupled to the 6,7- disubstituted 4'-chloroquinazoline moiety to furnish the final fluorine-18 labeled compounds. This methodology was used for the formation of several potential fluorine-18 labeled PET probes, derived from therapeutic agents such as erlotinib, gefitinib and ZD6474. [^18^F]ML01 (Fig. **4**) was one of the most studied compounds among these reversible labeled inhibitors. Its median inhibitory concentration (IC_50_) values in an enzyme-linked immunosorbent assay (ELISA), using A431-derived cell membranes that overexpress the receptor, and in intact A431 cells were 0.2 nM and 3.8 nM, respectively. [^18^F]-ML01 exhibited high specific binding and could accurately measure receptor content (B_max_) in intact A431 cells. In a study testing the kinetics of ML01 binding to EGFR, the association and dissociation-rate constants (K_on_, K_off_) were obtained, and a derived k_D_ value of 65 nM, defined as K_off_/K_on_, was calculated. According to the number of EGFR binding-sites per A431 cell [95-97], and assuming 10^9^ cells per gram of tumor, an estimated B_max_ value of 200 nM EGFR in the tumor tissue could be obtained, yielding a rather low binding potential (BP) of ~3. This could explain why although ML01 exhibited significant inhibitory potency and adequate specific binding to the receptor *in vitro*, its* in vivo* performance was less promising. Indeed, PET-imaging of A431 tumor-bearing mice following I.V. administration of [^18^F]-ML01 indicated that although tumor could be clearly detected, its visualization persisted for a narrow imaging time-frame of 8-12 min post injection. Thus, kinetic factors such as rapid dissociation from the receptor and elevated blood clearance rendered ML01 ineffective as a tracer for imaging the EGFR [94]. 

Gefitinib was labeled with fluorine-18 in a similar fashion to ML01 [92]. In addition, the radiolabeling of gefitinib with carbon-11 at the C-7 methoxy group has also been reported (Fig. **4**) [98]. The first biological evaluation performed with radiolabeled gefitinib was presented in 2003. The potential of the fluorine-18 labeled compound as an imaging agent was investigated by microPET studies; however, neither significant tumor uptake nor specific binding could be demonstrated [99]. These findings were supported by a recent and more detailed report [100] in which the microPET study did not show preferential activity uptake in tumors relative to surrounding skeletal muscle; furthermore, in all studied tumor types, the tumor: blood activity uptake ratio was < 1 during 2 h post injection of the tracer, and the obtained binding potential (BP) was similar in EGFR positive and negative xenografts. 

Although the above described labeled compounds are either identical to or derivatives of existing drugs, they have not yielded promising target-specific agents for PET tumor imaging since more prerequisites have to be fulfilled. It is of utmost importance that a tracer level administration of the imaging agent (as opposed to drug which can be administered up to the maximum tolerated dose) furnishes high and specific accumulation in the target tissue. This tumor uptake should be significantly higher than in the surrounding tissue to yield elevated signal: noise ratio which is mandatory for imaging. Thus, favorable pharmacokinetics and minimum levels of radiolabeled metabolites are equally required for adequate imaging. Up till now, anilinoquinazoline-based radiolabeled reversible inhibitors, although generally exhibiting high potential as imaging agents *in vitro*, they have not yielded adequate PET imaging of EGFR-overexpressing tumors in an animal model. This failure could be attributed to their elevated log P, fast blood clearance, rapid metabolism, and binding competition between manifold higher levels of intracellular ATP and the radioligand resulting in the rapid washout of the labeled inhibitor from the tumor. Subsequently, attempts have been made for the development of irreversible inhibitors of the EGFR as potential imaging agents (Fig. **4**) [101-111]. Out of the numerous developed labeled irreversible EGFR inhibitors, four were intensely studied as imaging agent candidates, i.e., [^11^C]-ML03, [^11^C] and [^18^F]-ML04, [^18^F]-FEQA and morpholino [^124^I]-IPQA. In this category of irreversible EGFR PET agent candidates, the carbon-11 labeled 6-acrylamido-4-(3,4-dichloro-6-fluoroanilino) quinazoline, ML03, (Fig. **4**) was the first to be synthesized and labeled [101, 103]. ML03 exhibited a remarkable inhibitory potency with an IC_50_ of 0.037 nM in a cell-free assay combined with sustained inhibition of the receptor in intact A431 cells; its specific binding to the receptor was demonstrated in a cell binding assay and also in tissue sections of liver and A431 tumors. However, its fast degradation *in vivo* in combination with low tumor activity uptake levels along with pronounced activity concentration in the liver, kidney and intestine, obtained in tumor-bearing rat biodistribution studies, rendered [^11^C]-ML03 ineffective as a tracer for imaging of the EGFR [99]. In 2003, the preparation and biological evaluation of an additional labeled acrylamido-anilinoquinazoline derivative, N-{4-[3'-[^18^F]fluoroethylphenyl)amino]-6-quinazolinyl}acrylamide ([^18^F]-FEQA) was reported [112]. A dynamic microPET scan was performed in A431 tumor-bearing mice in order to study tracer biodistribution. A rather low maximal tumor: blood activity uptake ratio of 0.12 was obtained shortly post administration of the tracer, which decreased over time. In contrast, a high activity uptake in metabolic and excretory organs was detected as inferred by the relatively high tissue: blood activity uptake ratio that was obtained at further time points in the gallbladder and bladder, indicating rapid clearance of the labeled compound probably *via* both renal and hepatobiliary routes. Thus, although the tumor could be visualized, high levels of background activity were observed, and more importantly, the EGFR specific tumor uptake was not evaluated. Another interesting EGFR-TK PET agent is the (E)-But-2-enedioic acid [4-(3-[^124^I]iodoanilino)-quinazolin-6-yl]-amide-(3-morpholin-4-yl-propyl)-amide (morpholino-[^124^I]-IPQA) (Fig. **4**) [110]. It has a unique potential as a PET agent since it has shown for the first time selective and irreversible binding to the ATP-binding site of the activated (phosphorylated), but not to the inactivated EGFR kinase, thus having the ability to discriminate *in vivo* between these two forms of the receptor. In dynamic PET scans of rats bearing two xenografts, a gradual accumulation of the tracer in A431 (EGFR-positive), but not in human chronic myeloid leukemia K562 (EGFR-negative) tumors, was observed. However, only a moderate activity uptake value in the positive xenograft of ~0.72 a% id/ g tumor, at 1 h post injection of the tracer was obtained. Although this approach indicates, to some extent, specific uptake, more convincing evidence such as direct *in vivo* blocking studies would be required. The major drawbacks of morpholino-[^124^I]-IPQA include low solubility, significant hepatobiliary clearance, and intestinal reuptake. 

**Fig. (6):**
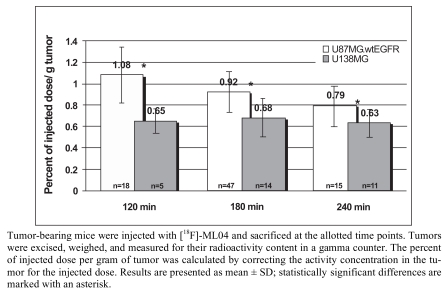
Preferential uptake of [18F]-ML04 in U87MG.wt EGFR over U138MG tumors.

**Scheme. (1):**
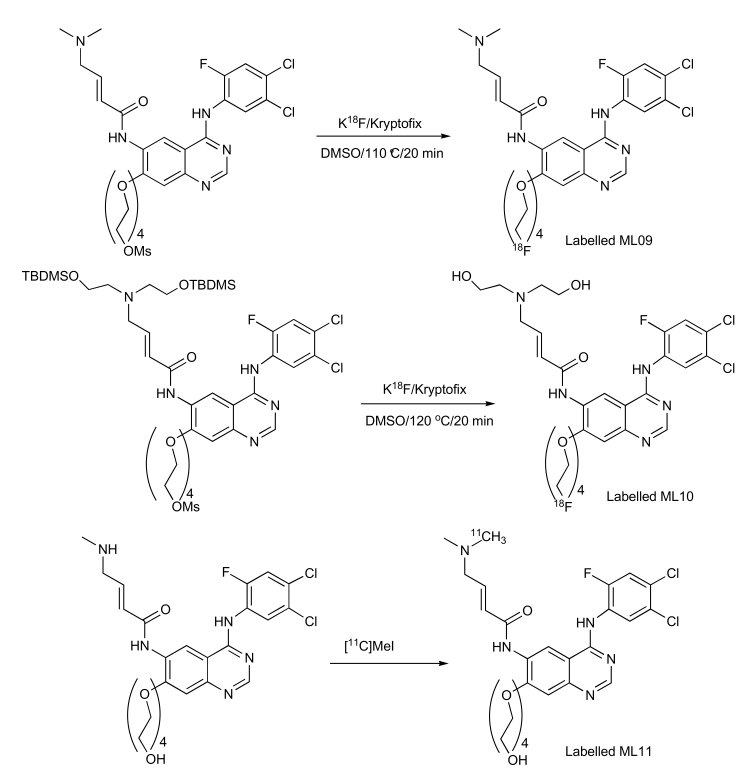
Labelling of ML09, ML10 and ML11.

Major progress with irreversible PET EGFR biomarkers has been achieved with the development of ML04 [109]. It demonstrated potent, irreversible inhibition of the EGFR in four human cell lines that express this receptor (Table **4**). The comparable inhibition levels obtained both immediately after and 8 h after removal of the inhibitor from the medium suggest that ML04 binds to the EGFR covalently and indeed inhibits activation of the receptor in an irreversible manner. This cell-based assay was originally introduced by Fry and colleagues as an additional supporting indicator of irreversible bonding [113]; later, this methodology has been routinely used as a reliable criterion of irreversible inhibition [106, 114, 115]. In addition, ML04 was found to selectively bind to the EGFR with a 250-fold inhibitory potency compared to other closely related tyrosine kinases with the exception of erbB2 which shares 80% homology with EGFR in its kinase domain (Table **4**). Following injection of the compound to U87MG.wt EGFR tumor-bearing mice (5 mg/kg i.v.), ML04 demonstrated significant inhibition of EGFR phosphorylation levels in tumors *in vivo* at different time points post administration. As indicated in Fig. (**5**), significant inhibition of phosphorylation persisted for at least 6 h post administration of the inhibitor indicating that, indeed, the compound reached the tumor and penetrated the cancer cells in sufficient quantity so as to bind to a relatively significant portion of the receptors for an extended period of time, as expected from irreversible inhibitors. The compound was successfully labeled either with carbon-11 on the dimethylamine moiety *via* C-11 methylation reaction using C-11 methyl iodide [105] or with fluorine-18 at the anilino moiety *via* a multistep radiosynthesis route [108]. In binding studies employing fluorine-18 ML04 and A431 cells, the compound demonstrated high specific binding of approximately 75% and could accurately measure the number of EGFR molecules per cell. In addition and in contrast to ML03, the previously labeled irreversible inhibitor, ML04 was found to be stable in blood either in ex vivo or *in vivo* studies. *In vivo* biodistribution of the radiolabeled compound revealed higher activity uptake in EGFR-positive tumors as compared to previously studied EGFR PET imaging agent candidates, and a remarkable tumor: blood and tumor: muscle activity uptake ratios of about 7 and 5, respectively, three hours following administration of the radiotracer (Table **5**). Nevertheless, only minor EGFR specific uptake of the compound was detected *in vivo* using EGFR-negative tumors or blocking studies as controls (Fig. **6**) [109]. A possible explanation of this observation could stem from the relatively high log P of ML04 which on the one hand, is required for cell penetration, while, on the other, it leads to high accumulation of the compound in the extracellular space of the tumor, resulting in high non-specific uptake. Therefore, derivatives of the compound such as ML09 [111], ML10 and ML11 (Fig. **4**) which exhibit lower log P, yet not too low to enable cell penetration, have been developed and labeled with fluorine-18 *via* one step radiosynthesis, Iodine-124 or carbon-11 (Scheme **1**) and are currently under investigation (unpublished results).

## SUMMARY

In conclusion, the EGFR has been recognized as one of the most promising targets for the treatment of cancer. Indeed, several drugs, either based on monoclonal antibodies targeting the extracellular domain of the receptor or small organic molecules targeting the tyrosine kinase domain, are FDA-approved and many others are in various stages of development. However, the therapeutic potential of targeting the EGFR remains to be refined and optimized. Quantitative PET molecular imaging, coupled with selective labeled biomarkers, may facilitate *in vivo* EGFR-targeted drug efficacy by noninvasively assessing expression of EGFR in tumor, guiding dose and regime by measuring target-drug binding, receptor occupancy, and duration of inhibition, as well as potentially detecting the existence of a primary or secondary mutation leading to either drug interaction or failure of EGFR recognition by the drug. Two approaches have been undertaken for the development of EGFR PET imaging agents: labeling monoclonal antibodies and small organic molecules. These labeled compounds were based on either approved or drugs in the process of development. In the case of labeled monoclonal antibodies, Cetuximab has provided the most encouraging results by exhibiting high specificity and high accumulation in tumor, however, its slow clearance from the bloodstream and metabolic tissues results in limited imaging contrast at early time points post injection. Therefore, the labeling of smaller antibody fragments may emerge in the near future. In the case of small organic molecules, the main hurdles to be overcome include excessive clearance from blood, nonspecific binding and inadequate pharmacokinetic properties. Even though some of these developed imaging agents were based on approved drugs, they did not yield adequate PET imaging. Based on these findings, it is evident that the properties of successful targeted drugs will not always lead to efficient tracer level imaging agents. Indeed, important features should be taken into consideration when designing optimal imaging agents including, affinity, selectivity, stability, suitable Log p, optimal blood clearance, low uptake in non target tissues, and high specific binding in target tissues. Future trends in this investigation will probably focus on the development of labeled EGFR affibodies targeting the extracellular domain and labeled small organic molecules directed toward the intracellular substrate binding site rather than the ATP binding site.

## Figures and Tables

**Fig. (5):**
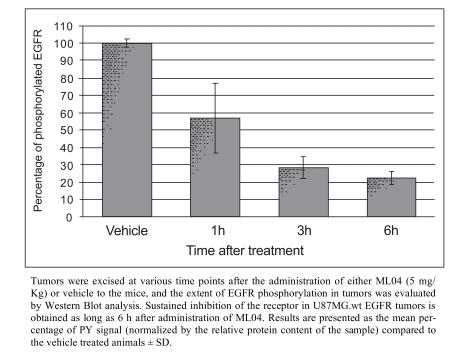
*In vivo* inhibition of phosphorylation of the EGFR in U87MG.wt EGFR xenografts is attained after administration of an excess of ML04 to mice.

**Table 1:** EGFR Expression in Solid Tumors [6, 116]

Tumor Type	Range of Tumors Expressing EGFR (%)	Tumor Type	Range of Tumors Expressing EGFR (%)
Head and neck	80–100	Prostate	40–80
Colorectal	25–77	Bladder	53–72
Pancreatic	30–50	Cervical	54–74
Lung	40–80	Ovarian	35–70
Esophageal	71–88	Breast	14–91
Renal cell	50–90	Glioblastoma	40–50

**Table 2:** Protein Tyrosine Kinases (PTKs) Targeted Drugs

Drug/*Type*	PTKs	Indication
Cetuximab (Erbitux)/ *mAb*	erbB1 (EGFR)	colorectal cancer, phase III for head/neck, pancreatic cancers and NSCLC, Phase II for HCC
Matuzumab (EMD 72000)/* mAb*	erbB1 (EGFR)	phase I/II for NSCLC, ovarian, pancreatic cancer
Panitumumab (ABX-EGF, Vectibix )/* mAb*	erbB1 (EGFR)	colorectal cancer, phase I for refractory solid Tumors
Erlotinib (Tarceva)*TK inhibitor*	erbB1 (EGFR)	NSCLC, pancreatic cancer, phase II for HCC
Gefitinib (Iressa)/* TK inhibitor*	erbB1 (EGFR)	NSCLC, phase I for HCC
EKB-569/* TK inhibitor*	erbB1 (EGFR)	phase II for advanced colorectal cancer and NSCLC
Lapatinib (Tykerb)/* TK inhibitor*	erbB1 (EGFR)/ erbB2	advanced metastatic breast cancer
Canertinib (CI-1033)*/TK inhibitor*	Pan-erbB	phase II for SCC, ovarian and metastatic breast cancer cancer
Trastuzumab (Herceptin)/* mAb*	erbB2	Breast cancer
Imatinib (Gleevec)/*TK inhibitor*	ABL, PGRFR, KIT	CML, CMML, CEL, GIST
Bevacizumab (Avastin)*/* mAb*	VEGF A	NSCLC, colorectal cancer
Semaxanib (SU5416)/* TK inhibitor*	VEGFR, EGFR, KIT	phase II for metastatic melanoma
Sunitinib (Sutent)/ *TK inhibitor*	VEGFR, KIT, PDGFR, Flt3	GIST, Renal cell carcinoma
Vatalanib/ *TK inhibitor*	VEGFR, PDGFR	phase III for colorectal and phase II for GIST, prostate and kidney cancer

**NSCLC**, non small cell lung cancer; **HCC**, hepatocellular cancer; **CML**, Chronic myeloid leukemia; **CMML**, Chronic myelomonocytic leukemia; **CEL**, Chroniceosinophilic leukemia; **GIST**, Gastrointestinal stromal tumor; **SCC**, Squamous cell carcinoma.

**Table 3:** Decay Characteristics of Radionuclides Used to Label Anti-EGFR mAbs

Isotope	T_1/2_	β ^-^MeV (max) (%)	β ^+^MeV (max) (%)	γ MeV (%)
^64^Cu	12.7 h	0.573 (39.6%)	0.655 (17.4%)	0.51 (34.8%)
^88^Y	108 d	-	-	0.898 (91%) 1.836 (100%)
^89^Zr	78.4 h	-	0.9 (22%)	0.511 (44%) 0.91 (99%) 1.71 (1%)
^111^In	67.4 h	-	-	0.173 (89%) 0.247 (94%)
^125^I	60.2 d	-	-	0.035 (7%)
^177^Lu	6.74 d	0.497 (90%) 0.385 (3%)	-	0.113 (2.8%) 0.208 (6.1%)

**Table 4:** ML04 is a Potent, Irreversible and Selective Inhibitor of the EGFR

Investigated Tyrosine Kinase (Cell Line)	IC_50_ Values in Intact Cells [nM] a		IC_50_ Values in a Cell-Free Kinase Assay [nM] a
	Immediately after Removal of the Inhibitor	Eight Hours after Removal of the Inhibitor	
EGFR (A431)	4-10 b	10-50 b	0.11 ± 0.08 b
EGFR (MDA-MB 468)	1-5	1-5	
EGFR (PC10)	10-50	10-50	
EGFR (NCI-H1975)	25	ND	
EGFR (DHER14)	4 b	ND	
HER2 (CSH12)	25-50 b	ND	ND
PDGFR (NIHPDGFR)	> 1000 b	ND	ND
VEGFR-2 (PAE/ KDR)	> 10,000	ND	ND
c-Src	ND		118 ± 26
IGF-1R	ND		> 15,000

a The median inhibitory concentrations (IC50) values were obtained from at least three independent experiments. Where applicable, results are presented as mean ± SD.

b Studies investigating the inhibitory potency toward the EGFR and related tyrosine kinases were carried out using either intact cells or a cell free kinase assay. ND: not determined.

**Table 5:** Biodistribution of [^18^F]-ML04 3 h Post Administration to U87MG.wt EGFR Tumor-Bearing Mice

Tissue	Percent of Injected Dose Per Gram of Organ (n=12)	Tumor: Tissue Activity Uptake Ratios (n=10)
Blood	0.17 ± 0.02	7.07 ± 1.11
Bone	0.40 ± 0.05	2.42 ± 0.12
Heart	0.33 ± 0.02	3.07 ± 0.20
Intestine	2.19 ± 0.14	0.46 ± 0.03
Kidneys	3.98 ± 0.25	0.25 ± 0.02
Liver	1.63 ± 0.09	0.59 ± 0.02
Lungs	6.11 ± 0.50	0.16 ± 0.01
Muscle	0.17 ± 0.03	6.07 ± 0.70
Skin	0.59 ± 0.04	1.62 ± 0.05
Spleen	2.70 ± 0.19	0.37 ± 0.02
Stomach	0.94 ± 0.09	1.13 ± 0.09
U87MG.wtEGFR tumor	0.99 ± 0.05	

## ABBREVIATIONS

C225: = Cetuximab
CML: = Chronic myelogenous leukemia
CT: = Computed tomography
DOTA: = 1,4,7,10- Tetraazacyclododecane-1,4,7,10-tetraacetic acid
DTPA: = Diethylenetriaminepentaacetic acid
ELISA: = Enzyme-linked immunosorbent assay
EGF: = Epidermal growth factor
EGFR: = Epidermal growth factor receptor
EOB: = End of bombardment
GIST: = Gastrointestinal stromal tumors
HCC: = Hepatocellular carcinoma
HER 2, 3, 4: = Human EGFR 2, 3, 4
IC_50_: = Median inhibitory concentration
%ID/g: = Percentage of the injected dose measured per gram of tissue or organ
IHC: = Immunohistochemistry
mM: = Millimolar
MRI: = Magnetic resonance imaging
nM: = Nanomolar
NSCLC: = Non small cell lung cancer
PDGFR: = Platelet-derived growth factor receptors
PET: = Positron emission tomography
PTKs: = Protein tyrosine kinases
PY: = Phosphotyrosine
RTK: = Receptor tyrosine kinase
SCCHN: = Small cell carcinoma of the head and neck
SPECT: = Single-photon emission computed tomography
TK: = Tyrosine kinase
VEGFR: = Vascular endothelial growth factor receptor

## References

[1] Parkin DM, Bray F, Ferlay J, Pisani P (2002). Global cancer statistics. CA Cancer J Clin.

[2] Ciardiello F (2000). Epidermal growth factor receptor tyrosine kinase inhibitors as agents. Drugs.

[3] Renhowe PA (2001). Growth factor receptor kinases in cancer. Ann Rep Med Chem.

[4] Rowinsky EK (2000). The pursuit of optimal outcomes in cancer therapy in a new age of rationally designed target-based anticancer agents. Drugs.

[5] Hubbard SR, Miller WT (2007). Receptor tyrosine kinases: mechanisms of activation and signaling. Curr Opin Cell Biol.

[6] Laskin JJ, Sandler AB (2004). Epidermal growth factor receptor: a promising target in solid tumours. Cancer Treat Rev.

[7] Yarden Y (2001). The EGFR family and its ligands in human cancer. Signaling mechanisms and therapeutic opportunities. Eur J Cancer.

[8] Grünwald V, Hidalgo M (2002). The epidermal growth factor receptor: a new target for anticancer therapy. Curr Probl Cancer.

[9] Kralovics RF, Passamonti AS, Buser SS, Teo R, Tiedt JR, Passweg A (2005). A gain-of-function mutation of JAK2 in myeloproliferative disorders. N Engl J Med.

[10] Levitzki A (2003). EGF receptor as a therapeutic target. Lung Cancer.

[11] Ritter CA, Arteaga CL (2003). The epidermal growth factor receptor-tyrosine kinase: a promising therapeutic target in solid tumors. Semin Oncol.

[12] Stern DF (2000). Tyrosine kinase signalling in breast cancer: ErbB family receptor tyrosine kinases. Breast Cancer Res.

[13] Futreal PA, Coin L, Marshall M, Down T, Hubbard T, Wooster R (2004). A census of human cancer genes. Nat Rev Cancer.

[14] Blume-Jensen P, Hunter T (2001). Oncogenic kinase signaling. Nature.

[15] Levitzki A, Mishani E (2006). Tyrphostins and other tyrosine kinase inhibitors. Ann Rev Biochem.

[16] Baselga J (2006). Targeting tyrosine kinases in cancer: the second wave. Science.

[17] Fry DW (1994). Protein tyrosine kinases as therapeutic targets in cancer chemotherapy and recent advances in the development of new inhibitors. Exp Opin Invest Drugs.

[18] Traxler P, Bold G, Buchdunger E, Caravatti G, Furet P, Manley P (2001). Tyrosine kinase inhibitors: from rational design to clinical trials. Med Res Dev.

[19] Hopfner M, Schuppan D, Scherubl H (2008). Growth factor receptors and related signaling pathways as targets for novel treatment strategies of hepatocellular cancer. World J Gastroenterol.

[20] Rodriguez J, Zarate R, Bandres E, Viudez A, Chopitea A, Garcia-Foncillas J (2007). Combining chemotherapy and targeted therapies in metastatic colorectal cancer. World J Gastroenterol.

[21] Giamas G, Stebbing J, Vorgias CE, Knippschild U (2007). Protein kinases as targets for cancer treatment. Pharmacogenomics.

[22] Sergina NV, Moasser MM (2007). The HER family and cancer: emerging molecular mechanisms and therapeutic targets. Trends Mol Med.

[23] Levitzki A (2003). Protein kinase inhibitors as a therapeutic modality. Acc Chem Res.

[24] Levitzki A (1992). Tyrphostins: tyrosine kinase blockers as novel antiproliferative agents and dissectors of signal transduction. FASEB J.

[25] Levitzki A, Gazit A (1995). Tyrosine kinase inhibition: an approach to drug development. Science.

[26] Imai K, Takaoka A (2006). Comparing antibody and small-molecule therapies for cancer. Nat Rev Cancer.

[27] Bennasroune A, Gardin A, Aunis D, Cremel G, Hubert P (2004). Tyrosine kinase receptors as attractive targets for cancer therapy. Crit Rev Oncol Hematol.

[28] Mass RD (2004). The HER receptor family: a rich target for therapeutic development. Int J Radiat Oncol Biol Phys.

[29] Mass RD, Press MF, Anderson S, Cobleigh MA, Vogel CL, Dybdal N (2005). Evaluation of clinical outcomes according to HER2 detecton by fluorescence *in situ* hybridizationo in women with metastatic breast cancer treated with trastuzumab. Clin Breast Cancer.

[30] Cunningham D, Humblet Y, Siena S, Khayat D, Bleiberg H, Santoro A (2004). Cetuximab monotherapy and cetuximab plus iri-notecan in irinotecan-refractory metastatic colorectal cancer. N Engl J Med.

[31] Bernier J (2006). Cetuximab in the treatment of head and neck cancer. Expert Rev Anticancer Ther.

[32] Chung KY, Shia J, Kemeny NE, Shah M, Schwartz GK, Tse A (2005). Cetuximab shows activity in colorectal cancer patients with tumors that do not express the epidermal growth factor receptor by immunohistochemistry. J Clin Oncol.

[33] Hoy SM, Wagstaff AJ (2006). Panitumumab: in the treatment of metastatic colorectal cancer. Drugs.

[34] Berlin J, Posey J, Tchekmedyian S, Hu E, Chan D, Malik I (2007). Panitumumab with irinotecan/leucovorin/5-fluorouracil for first-line treatment of metastatic colorectal cancer. Clin Colorectal Cancer.

[35] Cohenuram M, Saif MW (2007). Panitumumab the first fully human monoclonal antibody: from the bench to the clinic. Anticancer Drugs.

[36] Labianca R, La Verde N, Garassino MC (2007). Development and clinical indications of cetuximab. Int J Biol Markers.

[37] Bajetta E, Procopio G, Verzoni E, Catena L, De Dosso S, Platania M (2007). Renal cell cancer and sorafenib: skin toxicity and treatment outcome. Tumori.

[38] Graziani Y, Chayoth R, Karny N, Feldman B, Levy J (1982). Regulation of protein kinases activity by quercetin in Ehrlich ascites tumor cells. Biochim Biophys Acta.

[39] Graziani Y, Erikson E, Erikson RL (1983). The effect of quercetin on the phosphorylation activity of the Rous sarcoma virus transforming gene product *in vitro* and *in vivo*. Eur J Biochem.

[40] Akiyama T, Ishida J, Nakagawa S, Ogawara H, Watanabe S, Itoh N (1987). Genistein, a specific inhibitor of tyrosine-specific protein kinases. J Biol Chem.

[41] Onoda T, Iinuma H, Sasaki Y, Hamada M, Isshiki K, Naganawa H (1989). Isolation of a novel tyrosine kinase inhibitor, lavendustin A, from Streptomyces griseolavendus. J Nat Prod.

[42] Umezawa H, Imoto M, Sawa T, Isshiki K, Matsuda N, Uchida T (1986). Studies on a new epidermal growth factor-receptor kinase inhibitor, erbstatin, produced by MH435-hF3. J Antibiot (Tokyo).

[43] Imoto M, Umezawa K, Sawa T, Takeuchi T, Umezawa H (1987). *In situ* inhibition of tyrosine protein kinase by erbstatin. Biochem Int.

[44] Yaish P, Gazit A, Gilon C, Levitzki A (1988). Blocking of EGF-dependent cell proliferation of EGF receptor kinase inhibitors. Science.

[45] Gazit A, Yaish P, Gilon C, Levitzki A (1989). Tyrphostins I: synthesis and biological activity of protein tyrosine kinase inhibitors. J Med Chem.

[46] Shiraishi T, Owada MK, Tatsuka M, Yamashita T, Watanabe K, Kakunaga T (1989). Specific inhibitors of tyrosine-specific protein kinases: properties of 4-hydroxycinnamamide derivatives *in vitro*. Cancer Res.

[47] Shiraishi T, Owada MK, Tatsuka M, Fuse Y, Watanabe K, Kakunaga T (1990). A tyrosine-specific protein kinase inhibitor, alpha-cyano-3-ethoxy-4-hydroxy-5-phenylthiomethylcinnamamide, blocks the phosphorylation of tyrosine kinase substrate in intact cells. Jpn J Cancer Res.

[48] Druker BJ, Tamura S, Buchdunger E, Ohno S, Segal GM, Fanning S (1996). Effects of a selective inhibitor of the Abl tyrosine kinase on the growth of Bcr-Abl positive cells. Nat Med.

[49] Druker BJ, Talpaz M, Resta DJ, Peng B, Buchdunger E, Ford JM (2001). Efficacy and safety of a specific inhibitor of the BCR-ABL tyrosine kinase in chronic myeloid leukemia. N Engl J Med.

[50] Blanke CD, Corless CL (2005). State-of-the art therapy for gastrointestinal stromal tumors. Cancer Invest.

[51] Liu Y, Poon RT, Li Q, Kok TW, Lau C, Fan ST (2005). Both antiangiogeneis- and angiogenesis-independent effects are responsible for hepatocellular carcinoma growth arrest by tyrosine kinase inhibitor PTK787/ZK222584. Cancer Res.

[52] Steeghs N, Nortier JW, Gelderblom H (2007). Small molecule tyrosine kinase inhibitors in the treatment of solid tumors an update of recent developments. Ann Surg Oncol.

[53] Wiedmann N, Feisthammal J, Bluthner T, Tannapfel A, Kamenz T, Kluge A (2006). Novel targeted approaches to treatment biliary tract cancer: the dual epidermal growth factor receptor and ErbB-2 tyrosine kinase inhibitor NVP-AEE788 is more efficient than the epidermal growth factor receptor inhibitors gefitinib and erlotinib. Anticancer Drugs.

[54] De Mulder PH, Roigas J, Gillessen S (2006). A phase II study of sunitinib administered in a continuous daily regimen in patients with cytokine-refractory metastatic renal cell carcinoma. Proc Am Soc Clin Oncol.

[55] Fong TA, Shawver LK, Sun L, Tang C, App H, Powell TJ (1999). SU5416 is a potent and selective inhibitor of the vascular endothelial growth factor receptor (Flk-1/KDR) that inhibits tyrosine kinase catalysis, tumor vascularization, and growth of multiple tumor types. Cancer Res.

[56] Rosen P, Amado R, Hecht J (2000). A phase I/II study of SU5416 in combination with 5-FU/leucovorin in patients with metastatic colorectal cancer. Proc Ac Soc Clin Oncol.

[57] Wood JM, Bold G, Buchdunger E, Cozens R, Ferrari S, Frei J (2000). PTK787/ZK 222584, a novel and potent inhibitor of vascular endothelial growth factor receptor tyrosine kinases, impairs vascular endothelial growth factor-induced responses and tumor growth after oral administration. Cancer Res.

[58] Sridhar SS, Shepherd FA (2003). Targeting angiogenesis: a review of angiogenesis inhibitors in the treatment of lung cancer. Lung Cancer.

[59] Jung YD, Mansfield PF, Akagi M, Takeda A, Liu W, Bucana CD (2002). Effects of combination anti-vascular endothelial growth factor receptor and anti-epidermal growth factor receptor therapies on the growth of a gastric cancer in a nude mouse model. Eur J Cancer.

[60] Baselga J, Averbuch SD (2000). ZD1839 (‘Iressa’), as an Anticancer Agent. Drugs.

[61] Akita RW, Sliwkowski MX (2003). Preclinical studies with Erlotinib (Tarceva). Semin Oncol.

[62] Takano T, Ohe Y, Sakamoto H, Tsuta K, Matsuno Y, Tateishi U (2005). Epidermal growth factor receptor gene mutations and increased copy numbers predict gefitinib sensitivity in patients with recurrent non-small-cell lung cancer. J Clin Oncol.

[63] Paez JG, Janne PA, Lee JC, Tracy S, Greulich H, Gabriel S (2004). EGFR mutations in lung cancer: correlation with clinical response to gefitinib therapy. Science.

[64] Lynch TJ, Bell DW, Sordella R, Gurubhagavatula S, Okimoto RA, Brannigan BW (2004). Activating mutations in the epidermal growth factor receptor underlying responsiveness of non-small-cell lung cancer to gefitinib. N Engl J Med.

[65] Tsao MS, Sakurada A, Cutz JC, Zhu CQ, Kamel-Reid S, Squire J (2005). Erlotinib in lung cancer — Molecular and clinical predictors of outcome. N Engl J Med.

[66] Pao W, Miller V, Zakowski M, Doherty J, Politi K, Sarkaria I (2004). EGF receptor gene mutations are common in lung cancers from ‘‘never smokers’’ and are associated with sensitivity of tumors to gefitinib and erlotinib. Proc Natl Acad Sci USA.

[67] Riely GJ, Politi KA, Miller VA, Pao W (2006). Update on epidermal growth factor receptor mutations in non-small cell lung cancer. Clin Cancer Res.

[68] Pao W, Miller VA, Politi KA, Riely GJ, Somwar R, Zakowski MF (2005). Acquired resistance of lung adenocarcinomas to gefitinib or erlotinib is associated with a second mutation in the EGFR kinase domain. PLoS Med.

[69] Kobayashi S, Boggon TJ, Dayaram T, Jänne PA, Kocher O, Meyerson M (2005). EGFR Mutation and resistance of non–small-cell lung cancer to Gefitinib. N Engl J Med.

[70] Kwak EL, Sordella R, Bell DW, Godin-Heymann N, Okimoto RA, Brannigan BW (2005). Irreversible inhibitors of the EGF receptor may circumvent acquired resistance to gefitinib. Proc Natl Acad Sci USA.

[71] Yoshimura N, Kudoh S, Kimura T, Mitsuoka S, Matsuura K, Hirata K (2006). EKB-569, a new irreversible epidermal growth factor receptor tyrosine kinase inhibitor, with clinical activity in patients with non-small cell lung cancer with acquired resistance to gefitinib. Lung Cancer.

[72] Cohen MH, Williams GA, Sridhara R, Chen G, McGuinn WD Jr, Morse D (2004). United states food and drug administration drug approval summary: gefitinib (ZD1839; Iressa) tablets. Clin Cancer Res.

[73] Maione P, Gridelli C, Troiani T, Ciardiello F (2006). Combining targeted therapies and drugs with multiple targets in the treatment of NSCLC. Oncologist.

[74] Cai W, Niu G, Chen X (2008). Multimodality imaging of the HER-kinase axis in cancer. Eur J Nucl Med Mol Imaging.

[75] Weiner RE, Thakur ML (2005). Radiolabeled peptides in oncology: role in diagnosis and treatment. BioDrugs.

[76] Dziadziuszko R, Hirsch FR, Varella-Garcia M, Bunn Jr PA (2006). Selecting lung cancer patients for treatment with epidermal growth factor receptor tyrosine kinase inhibitors by immunohistochemistry and fluorescence *in situ* hybridization. Why, when, and how? Clin Cancer Res.

[77] Mishani E, Abourbeh G (2007). Cancer molecular imaging: radionuclide-based biomarkers of the Epidermal Growth Factor Receptor (EGFR). Curr Topics Med Chem.

[78] Goldenberg A, Masui H, Divgi C, Kamrath H, Pentlow KS, Mendelsohn J (1989). Imaging of human tumor xenografts with an indium-111-labeled anti-epidermal growth factor receptor monoconal antibody. J. Natl Cancer Inst.

[79] Wen X, Wu Q, Ke S, Ellis L, Charnsangavej C, Delpassand AS (2001). Conjugation with 111In-DTPA-Poly(ethylene glycol) improves imaging of anti-EGF receptor antobody C225. J Nucl Med.

[80] Fan Z, Masui H, Altas I, Mendelsohn J (1993). Blockage of epidermal growth factor receptor function by bivalent and monovalent fragments of C225 anti-epidermal growth factor receptor monoclonal antibodies. Cancer Res.

[81] Perk LR, Visser GW, Vosjan MJ, Stigter-van Walsum M, Tijink BM, Leemans CR (2005). (89)Zr as a PET surrogate radioisotope for scouting biodistribution of the therapeutic radiometals (90)Y and (177)Lu in tumor-bearing nude mice after coupling to the internalizing antibody cetuximab. J Nucl Med.

[82] Cai W, Chen K, He L, Cao Q, Koong A, Chen X (2007). Quantitative PET of EGFR expression in xenograft-bearing mice using (64)Cu-labeled cetuximab, a chimeric anti-EGFR monoclonal antibody. Eur J Nucl Med Mol Imaging.

[83] Li WP, Meyer LA, Capretto DA, Sherman CD, Anderson CJ (2008). Receptor binding, biodistribution and metabolism studies of ^64^Cu-DOTA-cetuximab, a PET imaging agent for epidermal growth factor receptor positive tumors. Cancer Biother Radiopharm.

[84] Nordberg E, Friedman M, Gostring L, Adams GP, Brismar H, Nilsson FY (2007). Cellular studies of binding, internalization and retention of a radiolabeled EGFR-binding affibody molecule. Nucl Med Biol.

[85] Friedman M, Nordberg E, Hoiden-Guthenberg I, Brismar H, Adams GP, Nilsson FY (2007). Phage display selection of Affibody molecules with specific binding to the extracellular domain of the epidermal growth factor receptor. Protein Eng Des Sel.

[86] Nordberg E, Orlova A, Friedman M, Tolmachev V, Stahl S, Nilsson FY (2008). *In vivo* and *in vitro* uptake of 111In, delivered with the affibody molecule (ZEGFR: 955)2, in EGFR expressing tumour cells. Oncol Rep.

[87] Friedman M, Orlova A, Johansson E, Eriksson TL, Hoiden-Guthenberg I, Tolmachev V (2008). Directed evolution to low nanomolar affinity of a tumor-targeting epidermal growth factor receptor-binding affibody molecule. J Mol Biol.

[88] Mulholland GK, Zheng Q-H, Winkle WL, Carlson KA (1997). Synthesis and biodistribution of new C-11 and F-18 labeled epidermal growth factor receptor ligands. J Nucl Med.

[89] Fredriksson A, Johnstrom P, Thorell JO, ven Heijne G, Hassan M, Eksborg S (1999). *In vivo* evaluation of the biodistribution of 11C-labeled PD153035 in rats without and with neuroblastoma implants. Life Sci.

[90] Mulholland GK, Winkle W, Mock BH, Sledge J (1995). Radioiodinated epidermal growth factor receptor ligands as tumor probels. Dramatic potentiation of binding to MDA-468 cancer cells in presence of EGF. J Nucl Med.

[91] Mattner F (2001). Radioiodinated epidermal growth factor receptor inhibitors for tumor imaging with SPECT. Quart J Nucl Med.

[92] Seimbille Y, Phelps ME, Czernin J, Silverman DHS (2005). Fluorine-18 labeling of 6,7-disubstituted anilinoquinazoline derivatives for positron emission tomography (PET) imaging of tyrosine kinase receptors: synthesis of 18F-Iressa and related molecular probes. J Label Comp Radiopharm.

[93] Mishani E, Bonasera TA, Rozen Y, Ortu G, Gazit A, Levitzki A (1999). Fluorinated EGFR-TK inhibitors-based tracers for PET. J Label Cpd Radiopharm.

[94] Bonasera TA, Ortu G, Rozen Y, Krais R, Freedman NM, Chisin R (2001). Potential (18)F-labeled biomarkers for epidermal growth factor receptor tyrosine kinase. Nucl Med Biol.

[95] Velikyan I, Sundberg AL, Lindhe OR, Ho¨glund AU, Eriksson O, Werner E (2005). Preparation and evaluation of ^68^Ga-DOTA-hEGF for visualization of EGFR expression in malignant tumors. J Nucl Med.

[96] Masui H, Castro L, Mendelsohn J (1993). Consumption of EGF by A431 Cells: Evidence for receptor recycling. J Cell Biol.

[97] Yang XD, Jia XC, Corvalan JRF, Wang P, Davis CG (2001). Development of ABX-EGF, a fully human anti-EGF receptor monoclonal antibody, for cancer therapy. Crit Rev Oncol Hematol.

[98] Wang J-Q, Gao M, Miller KD, Sledge GW, Zheng Q-H (2006). Synthesis of [^11^C]Iressa as a new potential PET cancer imaging agent for epidermal growth factor receptor tyrosine kinase. Bioorg Med Chem.

[99] DeJesus OT, Murali D, Flores LG, Converse AK, Dick DW, Oakes TR (2003). Synthesis of [F-18]-ZD1839 asa PET imaging agent for epidermal growth factor receptors. J Labelled Comp Radiopharm.

[100] Su H, Seimbille Y, Ferl GZ, Bodenstein C, Fueger B, Kim KJ (2008). Evaluation of [^18^F]gefitinib as a molecular imaging probe for the assessment of the epidermal growth factor receptor status in malignant tumors. Eur J Nucl Med Mol Imaging.

[101] Mishani E, Ben-David I, Rozen Y, Ortu G, Leviztki A (2001). Carbon -11 labeled irreversible inhibitor for mapping epidermal growth factor receptor tyrosine kinase (EGFR-TK). J Label Cpd Radiopharm.

[102] Ortu G, Ben-David I, Rozen Y, Freedman NM, Chisin R, Levitzki A (2002). Labeled EGFr-TK irreversible inhibitor (ML03).* In vitro* and *in vivo* properties, potential as PET biomarker for cancer and feasibility as anticancer drug. Int J Cancer.

[103] Ben-David I, Rozen Y, Ortu G, Mishani E (2003). Radiosynthesis of ML03, a novel positron emission tomography biomarker for targeting epidermal growth factor receptor *via* the labeling synthon: [C-11]Acryloyl chloride. Appl Rad Isotop.

[104] Shaul M, Abourbeh G, Jacobson O, Rozen Y, Laky D, Levitzki A (2004). Novel iodine-124 labeled EGFR inhibitors as potential PET agents for molecular imaging in cancer. Bioorg Med Chem.

[105] Mishani E, Abourbeh G, Rozen Y, Jacobson O, Lacy D, Ben David I (2004). Novel carbon-11 labeled 4-dimethylamino-but-2-enoic acid [4-(phenylamino)-quinazoline-6-yl]amides: potential PET bioprobes for molecular imaging of EGFR-positive tumors. Nucl Med Biol.

[106] Mishani E, Abourbeh G, Jacobson O, Dissoki S, Ben Daniel R, Rozen Y (2005). High-affinity epidermal growth factor receptor (EGFR) irreversible inhibitors with diminished chemical reactivities as positron emission tomography (PET)-imaging agent candidates of EGFR overexpressing tumors. J Med Chem.

[107] Vasdev N, Dorff PN, Gibbs AR, Nandanan E, Reid LM, O'Neil JP (2005). Synthesis of 6-acrylamido-4-(2-[18F]fluoroanilino)quinazoline: a prospective irreversible EGFR binding probe. J Labelled Comp Radiopharm.

[108] Dissoki S, Laky D, Mishani E (2006). Fluorine-18 labeling of ML04 – presently the most promising irreversible inhibitor candidate for visualization of EGFR in cancer. J Labelled Comp Radiopharm.

[109] Abourbeh G, Dissoki S, Jacobson O, Litchi A, Ben Daniel R, Laki D (2007). Evaluation of radiolabeled ML04, a putative irreversible inhibitor of the epidermal growth factor receptor, as a bioprobe for pet imaging of EGFR overexpressing tumors. Nucl Med Bio.

[110] Pal A, Glekas A, Doubrovin M, Balatoni J, Beresten T, Maxwell D (2006). Molecular imaging of EGFR kinase activity in tumors with 124I-labeled small molecular tracer and positron emission tomography. Mol Imaging Biol.

[111] Dissoki S, Aviv Y, Laky D, Abourbeh G, Levitzki A, Mishani E (2007). The effect of the [^18^F]-PEG group on tracer qualification of [4-(phenylamino)-quinazoline-6-yl]-amide moiety –- an EGFR putative irreversible inhibitor. Appl Rad Isotp.

[112] Waldherr C, Satyamurthy N, Toyokuni T, Wang S, Mellinghoff I, Tran C (2003). Evaluation of N-{4-[(3 '-[F-18]fluoroethylphenyl)amino]-6-quinazolinyl}acrylamide ([F-18]FEQA), a labeled tyrosine kinase inhibitor, for imaging epidermal growth factor receptor density. J Nucl Med.

[113] Fry DW, Bridges AJ, Denny WA, Doherty A, Greis KD, Hicks JL (1998). Specific, irreversible inactivation of the epidermal growth factor receptor and erbB2, by a new class of tyrosine kinase inhibitor. Proc Natl Acad Sci USA.

[114] Smaill JB, Showalter HD, Zhou H, Bridges AJ, McNamara DJ, Fry DW (2001). Tyrosine kinase inhibitors. 18. 6-Substituted 4-anilinoquinazolines and 4-anilinopyrido[3,4-d]pyrimidines as soluble, irreversible inhibitors of the epidermal growth factor receptor. J Med Chem.

[115] Tsou HR, Mamuya N, Johnson BD, Reich MF, Gruber BC, Ye F (2001). 6-Substituted-4-(3-bromophenylamino)quinazolines as putative irreversible inhibitors of the epidermal growth factor receptor (EGFR) and human epidermal growth factor receptor (HER-2) tyrosine kinases with enhanced antitumor activity. J Med Chem.

[116] Salomon DS, Brandt R, Ciardiello F, Normanno N (1995). Epidermal growth factor-related peptides and their receptors in human malignancies. Crit Rev Oncol Hematol.

[117] Hamoudeh M, Kamleh MA, Diab R, Fessi H (2008). Radionuclides delivery systems for nuclear imaging and radiotherapy of cancer. Adv Drug Deliv Rev.

[118] Pastorekova S, Zatovicova M, Pastorek J (2008). Cancer-associated carbonic anhydrases and their inhibition. Curr Pharm Des.

[119] Puttini M, Redaelli S, Moretti L, Brussolo S, Gunby RH, Mologni L (2008). Characterization of compound 584, an Abl kinase inhibitor with lasting effects. Haematologica.

